# Remission of cervicogenic dysphagia associated with biomechanical dysfunction following chiropractic therapy

**DOI:** 10.25122/jml-2023-0009

**Published:** 2023-05

**Authors:** Eric Chun-Pu Chu

**Affiliations:** 1. New York Chiropractic and Physiotherapy Centre, Hong Kong, China

**Keywords:** chiropractic, dysphagia, oropharyngeal, spinal biomechanics, spinal manipulative therapy, CSVL - Central Sacral Vertical Line, COG - Center of Gravity, ENT - Ear, Nose, and Throat, WHO - World Health Organization

## Abstract

Cervicogenic dysphagia is a complex condition that can arise from biomechanical dysfunction in the cervical spine. Conventional treatment outcomes are not always guaranteed. Chiropractic treatment is considered an alternative treatment for dysphagia, yet there is a lack of evidence supporting its effectiveness. We present the case of a 48-year-old male who had difficulty swallowing for eight months. He had a feeling of food stuck in his throat when eating hard food but without any pain when swallowing, and eventually, he could not swallow any dry food. He was diagnosed with dysphagia associated with an anxiety disorder and was treated with medication, but there was no improvement in his condition. A full-spine radiograph revealed biomechanical dysfunction, including reduced cervical lordosis and levoscoliosis of the upper thoracic spine. After nine months of conventional physiotherapy, the patient completely recovered from his symptoms, with significantly improved biomechanical parameters. This study highlights the potential mechanism of cervicogenic dysphagia and the effect of chiropractic treatment in managing it. Applying chiropractic treatment, including spinal manipulative therapy, instrument-assisted soft tissue manipulation, and mechanical traction, might bring a positive outcome for dysphagia patients with careful consideration.

## INTRODUCTION

Swallowing is a complex sensorimotor mechanism involving the coordination between different respiratory, oropharyngeal, and gastrointestinal tract muscles with the central nervous system. Normally, the tongue and oral muscles form a bolus that triggers involuntary muscle contractions in the pharynx and larynx to prevent the bolus from entering the lungs. The muscles of the tongue and pharynx then push the bolus down the esophagus and into the stomach. Dysfunction or weakness of the involved structures or muscles can result in dysphagia [1], affecting approximately 3% of the total population and more than 20% of people over 50 [2, 3]. Dysphagia is often considered an early sign of other conditions, such as neurological disorders, degeneration, musculoskeletal, inflammatory, psychological, or injuries [1, 4-6]. Cervicogenic dysphagia is characterized by difficulty swallowing caused by musculoskeletal and/or neural dysfunction in the cervical spine [4].

The underlying mechanisms of cervicogenic dysphagia are not yet fully understood. However, it is widely believed that the condition is primarily caused by a mechanical obstruction caused by a change in the position or stability of the cervical spine. This can occur due to trauma, degenerative changes, or biomechanical dysfunction. The altered position or instability of the cervical spine can compress or irritate the surrounding nerves and tissues, leading to a range of symptoms such as neck pain, difficulty in head rotation, and swallowing [2, 4, 5, 7].

Another possible mechanism of cervicogenic dysphagia is a disruption of the sensory feedback loop between the muscles of the cervical spine and the pharynx. The cervical spine plays a crucial role in the coordination of the swallowing process by sending sensory information to the brainstem. This feedback loop helps to synchronize the movements of the muscles in the neck and throat to ensure efficient and safe swallowing. Any disruption of this sensory feedback loop can result in coordination deficits and difficulty swallowing [1, 8].

Conservative treatments, such as postural modification, are often used in managing cervicogenic dysphagia, but the outcome is not guaranteed [2, 9]. Chiropractors are healthcare practitioners who frequently see patients with neuromusculoskeletal issues and often use spinal manipulative therapy (SMT) to improve cervical spine mobility and stability [10]. This treatment might benefit such patients, yet there is limited evidence regarding its effectiveness and safety.

Given the possibility of cervicogenic dysphagia, patients might complain about neck discomfort and seek chiropractic care. We present the case of a male with cervicogenic dysphagia and persistent neck pain who improved with multimodal chiropractic care.

## Case Presentation

### Patient information

A 48-year-old man, a non-smoking engineering professor, complained of gradually increasing difficulty in swallowing for 8 months. The patient first experienced a grabbing sensation in the throat and was reviewed at the outpatient department of a local hospital. He had a feeling of food stuck in his throat when eating hard food but did not experience any pain. He also described a 5-year history of neck pain and tightness in the upper trapezius region during office work. Although any food or liquid consumed never came out of the nose, his symptoms gradually worsened, and he eventually could not swallow any dry food, such as cake or bread for breakfast. He denied any family history of a similar condition.

His ear, nose, and throat (ENT) and cardiovascular examinations were reviewed at the local ENT hospital. ENT, respiratory and cardiovascular pathologies were ruled out, and his esophagogastroduodenoscopy was normal. He was diagnosed with dysphagia associated with an anxiety disorder at the psychiatry department. He was treated with physiotherapy and medications, including Lexapro (10 mg), Lyrica (75 mg), and Lexotan (1.5 mg).

Owing to an unsatisfactory response to therapy for 3 months, he could only eat chopped noodles and porridge and was unable to dine out at any restaurant. Difficulty swallowing negatively impacted his quality of life, and he lost over 10 kg in 5 months. Three months later, he was unable to consume thick liquids and sometimes even choked on his saliva during swallowing. The patient was then admitted to the emergency department.

The ENT specialist ruled out intracranial/neck pathology such as cervical facet syndrome, anterior osteophytes, disc herniation, idiopathic skeletal hyperostosis, and inflammatory diseases (rheumatoid arthritis, osteoarthritis) based on magnetic resonance imaging of the brain and soft tissue of the neck. The patient was discharged and followed a diet of porridge alone for 2 months but without any improvement in his condition. He searched for alternative therapies online and found similar case reports from his PubMed searches. He sought chiropractic therapy as an alternative therapy.

### Clinical Findings

Upon physical examination, the patient was found to have a chin-down posture. A physical examination of the ENT performed by an on-site ENT specialist did not reveal any pathology. The cervical range of motion was restricted to 15° passive extension (normal >60°) and 35° bilateral rotation (normal >80°). The intensity of the neck pain was rated as 7 out of 10 on a 10-point numeric rating scale.

The bilateral longus colli, longus capitis, pectoral muscles, scalene, upper trapezius, sternocleidomastoid, and mid-back paraspinal muscles were moderately hypertonic. Spinal motion palpation identified restricted segments at C2/3, C5/6, C6/C7, T1/2, T4/5, and thoracolumbar junction.

**Figure 1.**
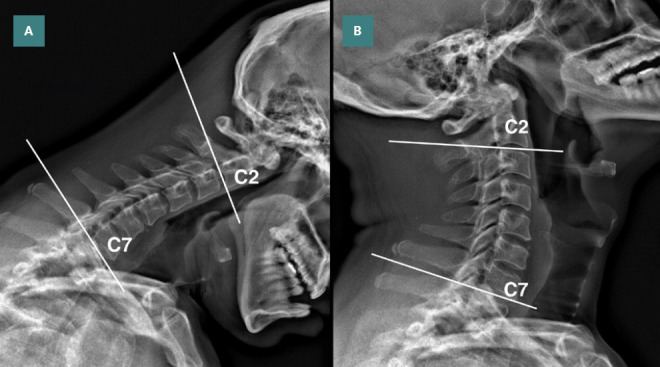
Flexion and extension views of cervical radiography. A: Cobb angle was measured at 9° in flexion and B: 16° in extension from the bottom of C2 to the bottom of C7 before treatment

**Figure 2.**
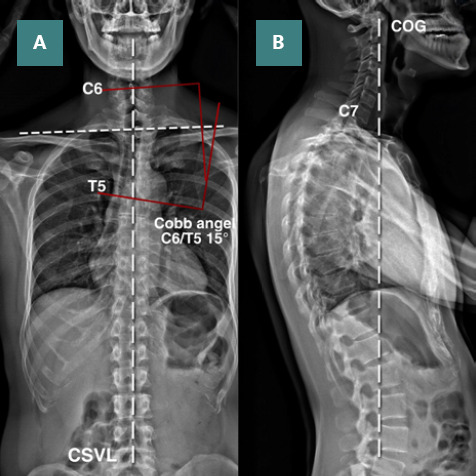
Biomechanical assessment in standing position with full spine radiography. A: Postural radiography demonstrated shoulder imbalance in the anterior forehead view. C-shaped cervicothoracic curve was measured at a Cobb angle of 15°. The central sacral vertical line (CSVL) shows the global axis. B: The center of gravity line (COG) should fall at the vertebral body of C7. If the line falls forward of C7, it indicates a forward head posture

Cervical radiographs in flexion and extension revealed reduced motion (Figure 1 A-B). Full-spine radiography revealed reduced cervical lordosis, forward head posture, and mild levoscoliosis of the lower cervical and upper thoracic spines (Figure 2 A-B). The World Health Organization (WHO) quality of life score was 47. Based on the physical presentation and radiological findings, the patient was diagnosed with cervicogenic dysphagia associated with biomechanical dysfunction suggestive of cervical neural impaction.

### Treatment and patient prognosis

The chiropractic therapy included spinal manipulative therapy of the cervical and thoracic regions, along with instrument-assisted soft tissue manipulation (gua sha) to release muscular tightness and spinal motions. Therapy sessions were conducted 12 times for the first month. Subsequently, mechanical traction (Robomax Series, WIZ Medical, Korea) was additionally applied at the lower cervical spine in the second month to decompress neural impaction. The therapy sessions were reduced to 8 times in the second month. The patient reported that his symptoms gradually improved over the third week of therapy, as he noticed he could take lunch with his colleagues. He started eating toast and eggs as normal breakfast in the fourth week and could eat steak at dinner during the eighth week.

**Figure 3.**
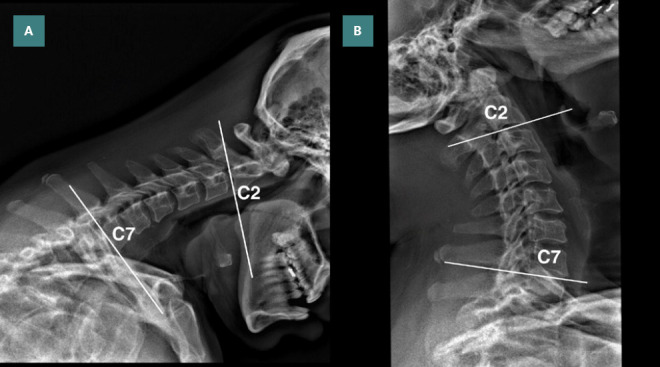
Post-treatment flexion and extension views of cervical radiographs at ninth month of treatment. A: Cobb angle was improved by 27° in flexion, and B: 28° in extension from the bottom of C2 to the bottom of C7

**Figure 4.**
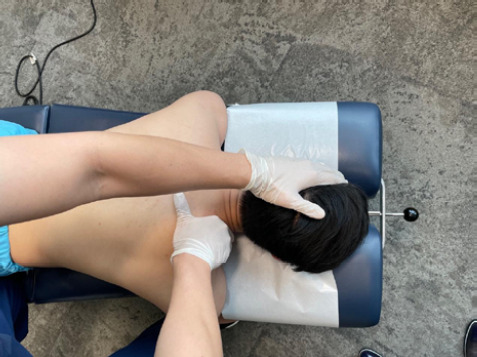
Demonstration of spinal manupulation by a chiropractor

Due to the COVID lockdown, he did not attend any therapy after the eighth week but was able to slowly cease taking all medications in the third month. The resolution of the symptoms was reported in the sixth month. Repeated radiographs at the 9-month follow-up examination showed improvement in his cervical flexion and extension (Figure 3 A-B) and biomechanical parameters, including cervical curvature, forward head posture, and cervicothoracic scoliosis (Figure 4). The patient reported occasional psychological stress when swallowing pills; however, this symptom was alleviated, and the patient could resume a regular diet without experiencing any discomfort. Subsequently, there was a significant improvement in the patient's quality of life, with the WHO quality of life score increasing from 47 to 98. The Eating Assessment Tool score improved from 29 to 2, indicating a positive outcome.

## Discussion

This case report describes a 48-year-old man who complained of difficulty swallowing for 8 months. The patient had a history of neck pain and tightness in the upper trapezius region during office work. His symptoms gradually worsened, and he eventually could not swallow any dry food. Despite multiple consultations and treatments, his condition did not improve until he sought chiropractic therapy. Based on the physical presentation and radiological findings, he was diagnosed with cervicogenic dysphagia associated with biomechanical dysfunction suggestive of cervical neural impaction.

We suggested that the patient’s cervicogenic dysphagia was associated with cervical biomechanical dysfunction. The cervical spine plays an important role in the movement of the head and neck, and it is involved in the coordination of swallowing. The muscles and nerves in the neck region are responsible for controlling the movement of the larynx, hyoid bone, and pharynx during swallowing [11]. The underlying mechanism of cervicogenic dysphagia is thought to be related to biomechanical dysfunction in the cervical spine [2, 4, 5, 7]. Biomechanical dysfunction can be caused by poor posture, trauma, degenerative changes, or muscular imbalances [12]. These factors can result in restricted joint mobility, muscle hypertonicity, and nerve impingement, which can affect the coordination of swallowing [11-13].

In the current case, the patient had a history of neck pain and tightness in the upper trapezius region during office work. Physical examination revealed a restricted cervical range of motion, moderate hypertonicity of the cervical and thoracic muscles, and spinal motion palpation identified restricted segments. Radiological findings showed reduced cervical lordosis, forward head posture, and mild levoscoliosis of the lower cervical and upper thoracic spines. These findings suggest that the patient had biomechanical dysfunction in the cervical spine, which may have contributed to his dysphagia [13-15].

Additionally, two similar cases involving a 53-year-old and a 70-year-old female diagnosed with dysphagia and cervical spondylosis presented to a chiropractor [4, 16]. Similar to other documented cases, these patients experienced neck symptoms and responded poorly to conservative treatments like medication, physical therapy, or acupuncture. Despite the difference in the underlying cause of the current case compared to other cases, all patients exhibited improvement after receiving chiropractic care. Based on these observations, we believe chiropractic treatment is beneficial in managing dysphagia.

Chiropractors commonly employ spinal manipulative therapy (SMT) and instrument-assisted soft tissue manipulation (gua sha) as techniques for treating musculoskeletal conditions [10]. SMT involves the use of high-velocity, low-amplitude thrusts to restore joint mobility and reduce muscle hypertonicity [17]. Gua sha involves using a smooth-edged instrument to apply pressure to the skin, which can help break up fascial adhesions and reduce pain and inflammation [18].

In cervicogenic dysphagia, SMT and gua sha were utilized to address muscular tightness and improve spinal motion [17,18]. It is suggested that SMT might provide a pain-reduction effect and improve spinal motion, which can help to reduce nerve impingement and restore joint mobility. Enhanced joint mobility enables the surrounding muscles to function more efficiently, alleviating tension in the gastrointestinal muscles and ultimately improving swallowing function [17, 19-21].

Similarly, gua sha can reduce pain and improve mobility in the affected tissues. By breaking up fascial adhesions, gua sha helps relieve muscle tension and enhances blood circulation in the targeted area. This increased blood flow assists in reducing inflammation and promoting healing, potentially leading to improved swallowing function [18].

In addition to SMT and gua sha, mechanical traction was applied at the lower cervical spine to decompress neural impingement. Mechanical traction involves using a device to gently stretch the spine, which can help reduce pressure on the nerves and promote healing. By reducing neural impingement, mechanical traction can help improve the coordination of swallowing and reduce symptoms of dysphagia [22].

Overall, these treatments aim to restore joint mobility, reduce muscle hypertonicity, and relieve nerve impingement, which can improve the coordination of swallowing and reduce symptoms of dysphagia. By improving the functionality of the musculoskeletal system, these interventions can facilitate healing and restore the normal function of swallowing.

Chiropractic treatment is generally considered safe, and adverse effects are rare [23]. However, it is important to note that dysphagia can be a sign of other pathologies [1, 4-6]. Chiropractors must exercise caution and remain vigilant for red flags in patients to prevent contraindications of treatment [24].

The current case report presents several limitations that should be acknowledged. Firstly, due to the patient's prior management at an outside hospital, obtaining previous ENT and physiotherapist follow-up information was impossible, including esophagogastroduodenoscopy findings and details of the previous physical therapy plan. No muscle activity testing was conducted to examine the patient's muscle activity condition. This case might not be widely generalizable, as the scope of chiropractic practice varies by location, and other chiropractors may be unable to arrange radiological investigations.

## Conclusion

Cervicogenic dysphagia is a complex condition that can be caused by various mechanisms. The dysfunction of the cervical spine, which may result from the hypertonicity of the muscles and the restriction of the range of motion, can lead to biomechanical dysfunction and the development of cervicogenic dysphagia. Multimodal chiropractic treatment might provide beneficial outcomes in managing cervicogenic dysphagia. However, healthcare providers must be mindful of potential red flags in dysphagia patients to prevent contraindications.
